# Depletion and Reversal of Hepatocellular Carcinoma Inducing CTL through ER Stress-Dependent PERK-CHOP Signaling Pathway

**DOI:** 10.1155/2022/6413783

**Published:** 2022-10-10

**Authors:** Mengnan Guo, Wei Wang, Wen Bai, Zekun Bai, Weixi Chen, Yali Su, Jinghua Wu

**Affiliations:** Tangshan Maternal and Child Health Care Hospital, North China University of Science and Technology, Tangshan 063000, Hebei, China

## Abstract

**Aims:**

In this report, it was investigated that hepatoma cells can cause downregulation of cytotoxic T lymphocyte (CTL) function and tea polyphenols (TPs) can reverse downregulation of CTL function.

**Methods:**

The expression of GRP78, PD-1, and TIM-3 was detected by western blotting in CTLL-2 cocultured with Hepa1-6 cells. Moreover, perforin (PRF1) and granzyme B (GzmB) protein levels and ER morphology were examined by ELISA and TEM, respectively. After 4-phenylbutyric acid (4-PBA) or tunicamycin (TM) treatment, programmed cell death protein 1 (PD-1), and mucin domain 3 (TIM-3), PRF1, and GzmB were measured by western blotting and ELISA. After sh-CHOP or GSK2656157 (PERK inhibitor) stimulation, the activation of the PERK-CHOP pathway was detected in CTLL-2 cells. Finally, changes in PD-1, TIM-3, PRF1, and GzmB levels were detected to verify the reversal of CTL depletion by TP.

**Results:**

The expression of GRP78, PD-1, and TIM-3 clearly increased, and swelling was observed for the endoplasmic reticulum (ER) in CTLL-2 cells cocultured with hepatoma cells. Concurrently, the levels of PRF1 and GzmB decreased. CTLL-2 depletion was induced after stimulation with TM and differed from 4-PBA stimulation. Treatment with sh-CHOP or GSK2656157 caused a decrease in PD-1 and TIM-3 expression, whereas the expression of PRF1 and GzmB clearly increased. After adding TP, the function of CTLs increased markedly.

**Conclusion:**

Hepatoma cells induced the depletion of CTLs through the ER stress PERK-CHOP pathway, and TP reversed this depletion by downregulating ER stress.

## 1. Introduction

Hepatocellular carcinoma (HCC) is one of the most lethal cancers in the world [1] that seriously endangers human health. The occurrence and development of liver cancer are extremely complex, and its etiology remains unclear. Multiple studies have found that the tumor microenvironment is closely related to the progression of tumors. [2, 3] CTLs, which are cells that play a role in eliminating tumors, are in a state of functional depletion because of long-term stimulation by tumor antigens. [4–6] However, the specific cellular depletion and regulation mechanisms remain unclear.

ER stress plays an essential role in the maturation, activation, function and/or depletion, death, and apoptosis of cells, including immune cells, and can also alter disease outcomes. [7–9] The occurrence of ER stress can be mediated through different pathways, and it has been shown that apoptosis of HCC cells is induced through the protein kinase RNA-like endoplasmic reticulum kinase (PERK)/C/EBP homologous protein (CHOP) pathway and affects the progression of HCC under ER stress conditions. [10–12] However, the role and mechanism of ER stress in inducing CTLs in liver cancer have not been reported.

The treatment of cancer by reversing the loss of T cell function is a hot topic in current cancer research and primarily involves blocking immune checkpoint PD-1, TIM-3, and other inhibitory pathways [13]. However, this method has significant toxic effects and causes cell depletion after a period of action. TP have anticancer, antimutagenic, antioxidant, antiaging, and antibacterial effects, [14,15] and these compounds may reverse the depletion of CTLs to enhance the removal of tumor cells. This potential activity of TP is a current research focus.

Therefore, in this study, we further clarify the role of ER stress and its pathways in regulating CTLs and their reversal to facilitate new research concepts and methods for the precise diagnosis and treatment of liver cancer.

## 2. Materials and Methods

### 2.1. Cell Lines and Culture Conditions

Mouse T lymphocyte (CTLL-2) cells were purchased from BeNa Culture Collection, and CTLL-2 cells were cultured in RPMI-1640 medium (C11875500, Gibco, China) supplemented with 10% fetal bovine serum (FBS, BI, USA), 100 U/mL penicillin, and 100 *μ*g/mL streptomycin (P1400, SolarBio, China) at 37°C and 5% CO_2_. Mouse hepatoma cells Hepa1-6 were purchased from ATCC, and the cells were cultured in DMEM-high glucose medium (C11995500, Gibco, China) containing 10% FBS, 100 U/mL penicillin, and 100 *μ*g/mL streptomycin.

### 2.2. Cell Coculturing

CTLL-2 cells were activated by adding the anti-CD3 antibody (kx10-3A, KEXIN, China), anti-CD28 antibody (kx10-28A, KEXIN, China), and interleukin-2 (IL-2, AF-212-12, PeproTech, USA). Activated CTLL-2 cells (1.5 × 10^6^/well) were then cocultured with Hepa1-6 (1 × 10^6^/well) cells. After different treatments, cells were collected for western blotting analysis. The morphology of the ER was observed by electron microscopy. The supernatant was used for effector molecule determination in cells.

### 2.3. Reagents

DMSO was purchased from Solarbio (D8371, China). TM was purchased from Abcam (ab120296, UK). 4-PBA was purchased from Sigma-Aldrich (P21005-25G, China). PERK selective inhibitor GSK2656157 was purchased from MedChemExpress (HY-13820, China), and tea polyphenols were obtained from Meilunbio (MB5041-1, China).

### 2.4. Cell Transfection

The specific shRNA of CHOP was designed and provided by GeneCopoein (MSH091766-LVRU6GP, China). Before transfection, the serum-containing medium was aspirated, and a serum-free medium was added. After harvesting, the cells were resuspended in Opti-MEM. The cell density was adjusted to 3 × 10^6^/20 *μ*L Opti-MEM. The cells were mixed with 5 *μ*g of CHOP-silencing plasmid. A voltage of 200 mV was selected for transfection. The pulse time was 400 ms with 10 repetitions.

### 2.5. Western Blotting Analysis

Cells were collected and lysed in the whole-cell lysate (containing phenylmethylsulfonyl fluoride and a phosphatase inhibitor). Equal amounts of cell lysates were separated by 10% SDS-PAGE and transferred to PVDF membranes. After blocking in 5% skimmed milk for 1 h at 37°C, membranes were incubated overnight at 4°C with the following primary antibodies: anti-GRP78 (AF5366, Affinity, China, 1 : 1000), anti-PERK (ab229912, Abcam, UK, 1 : 1000), anti-phospho-PERK (p-PERK, DF7576, Affinity, China, 1 : 2000), anti-CHOP (BF8081, Affinity, China, 1 : 1000), anti-programmed cell death receptor 1 (PD-1, ab214421, Abcam, UK, 1 : 1000), and anti-mucin domain 3 (TIM-3, D3M9R, Cell Signaling Pathway, USA, 1 : 1000), and anti-GAPDH (BM1623, Boster Biological Technology, China, 1 : 5000) was used as a control. Following the primary incubation, the membranes were incubated with horseradish peroxidase-conjugated goat antimouse IgG secondary antibody (ZJ2020, Biygot, China, 1 : 10000) at room temperature for 2 h. After washing the membrane with TBST for 45 min, the protein signal was detected using ECL Plus Hypersensitive Luminescence solution (AR1197, Boster, China).

### 2.6. Enzyme-Linked Immunosorbent Assay (ELISA)

The cell culture medium was transferred to a sterile centrifuge tube and centrifuged at 4°C. The supernatant was aliquoted into a centrifuged tube and used for the concentration-dependent detection of PRF1 (E-EL-M0890c, ElabScience, China) and GzmB (ELMO003, Boxbio, China) in strict accordance with the manufacturer's instructions.

### 2.7. Transmission Electron Microscopy (TEM)

TEM was used to assess the ER structure of CTLL-2 cells subjected to different treatments. After harvesting cells by centrifugation for 3 min, the supernatant was removed, and 2.5% glutaraldehyde was added for fixation. The sample was rinsed with 0.1 M phosphate buffer three times for 15 min each. Dehydration was performed with different concentrations of alcohol for 20 min and 100% acetone for 15 min twice for embedding. The embedded cells were sectioned and placed, and the images were observed using a TEM (H-7650, Japan).

### 2.8. Statistical Analysis

Data were analyzed by SPSS 19.0 software and expressed as mean ± SD. Differences were compared by one-way analysis of variance. Values of *P* < 0.05 were considered statistically significant.

## 3. Results

### 3.1. Depletion of CTLs Is Induced by Hepatoma Cells

**Figure 1:**
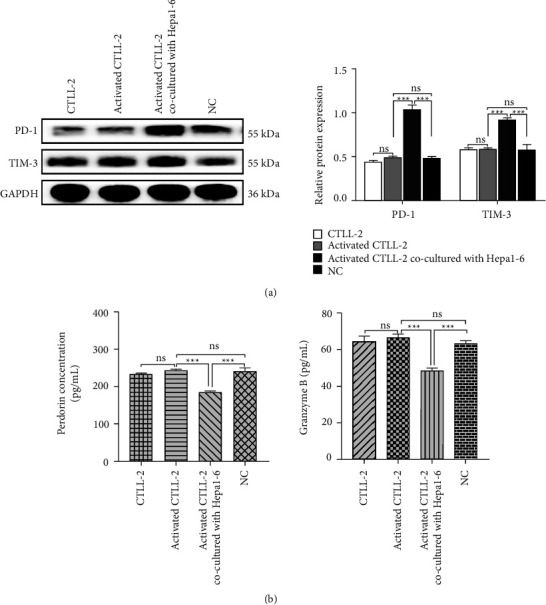
Function of CTLs was induced by hepatoma cells. (a) CTLL-2 cells were cocultured with hepatoma cells. PD-1 and TIM-3 expressions were determined by western blotting analysis. (b) PRF1 and GzmB expression was analyzed by ELISA. Data are presented as the mean ± SD. ^*∗∗∗*^*P* < 0.001, ns indicates no statistical significance.

We performed western blotting and ELISA analysis to evaluate the changes in cell function after CTLs were stimulated by hepatoma cells (Figure 1). The expression levels of inhibitory receptors PD-1 and TIM-3 were upregulated significantly (Figure 1(a)), whereas the secretion of effector molecules PRF1 and GzmB increased (Figure 1(b)) in CTLL-2 cells cocultured with Hepa1-6 cells. The results demonstrated that hepatoma cells induce CTLL-2 cells depletion.

### 3.2. Induction of ER Stress in CTLs by Hepatocellular Carcinoma Cells

**Figure 2:**
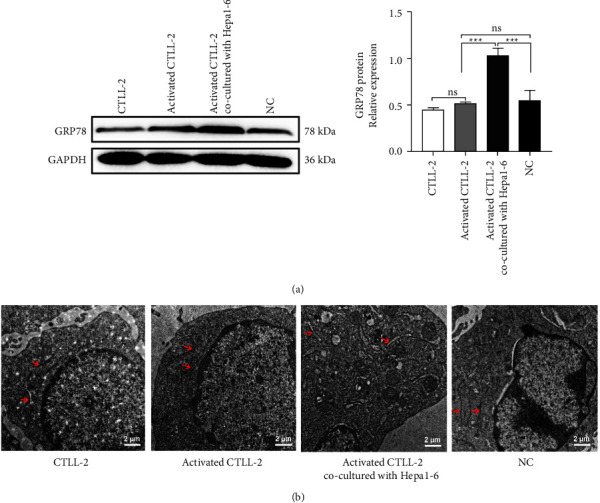
ER stress in CTLs was induced by hepatoma cells. (a) After coculturing hepatoma cells with CTLL-2 cells, GRP78 protein expression was detected by western blotting. (b) ER morphology was detected by electron microscopy (scale bar = 2 *μ*m). Data are presented as the mean ± SD. ^*∗∗∗*^*P* < 0.001, ns indicates no statistical significance.

ER stress plays an important role in regulating immune cell functions in the tumor microenvironment. [16] Thus, we tested the expression of ER stress-related proteins in CTLL-2 cells. The results showed that the level of GRP78 expression was upregulated (Figure 2(a)), and marked swelling of ER morphogenesis was observed after coculturing Hepa1-6 and CTLL-2 cells (Figure 2(b)).

### 3.3. Endoplasmic Reticulum Stress Induces CTLs Depletion

**Figure 3:**
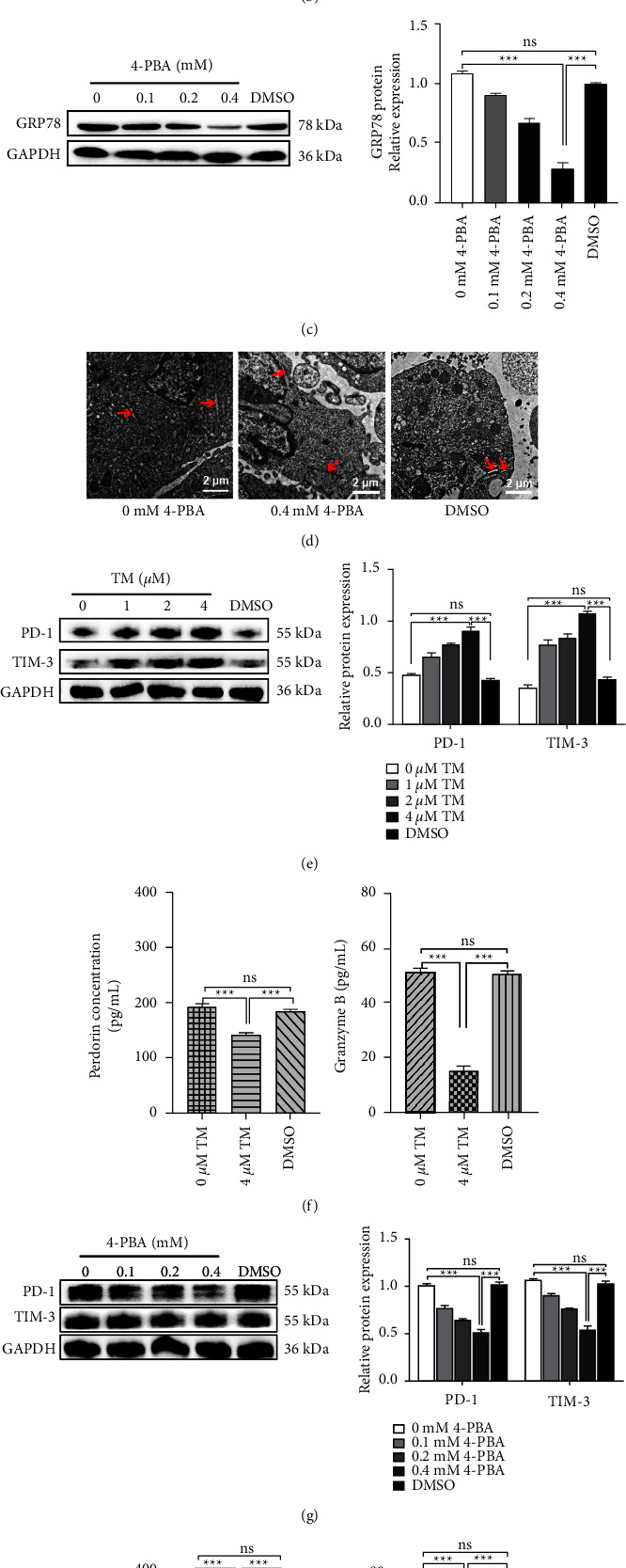
Activation of ER stress induces CTLs depletion. (a) CTTL-2 cells were cocultured with Hepa1-6 cells for one day. Different concentrations of TM (0, 1, 2, and 4 *μ*M) were added to CTTL-2 cells and cultured for 1 day. Western blotting analysis was used to determine GRP78 levels. (b) Cells were treated with TM (0 and 4 *μ*M), and changes in ER morphology were analyzed by electron microscopy (scale bar = 2 *μ*m). (c) CTTL-2 cells were cocultured with Hepa1-6 cells for one day. Different concentrations of 4-PBA (0, 0.1, 0.2 and 0.4 mM) were added to CTTL-2 cells and cultured for one day. The expression of GRP78 was analyzed by western blotting. (d) Cells were treated with 4-PBA (0 and 0.4 mM), and ER morphology was examined by electron microscopy. (e) CTLL-2 cells were treated with TM, and western blotting was used to analyze PD-1 and TIM-3 expression. (f) ELISA analysis of PRF1 and GzmB expression. (g) CTLL-2 cells were treated with 4-PBA, and PD-1 and TIM-3 expression levels were measured by western blotting. (h) ELISA was used to detect the expression of PRF1 and GzmB. Data are presented as the mean ± SD. ^*∗∗∗*^*P* < 0.001, ns indicates no statistical significance.

To test whether T cell depletion is caused by ER stress, we added TM, an ER stress activator, and found that the expression of GRP78 was upregulated significantly in CTLL-2 cells (Figure 3(a)). Swelling of the endoplasmic reticulum was also observed (Figure 3(b)). In contrast, treating CTLL-2 cells with 4-PBA, an inhibitor of ER stress, resulted in downregulation of GRP78 protein expression (Figure 3(c)) and restoration of normal ER morphology (Figure 3(d)). We next activated or inhibited ER stress to further verify changes in the cell function. Here, adding TM to the CTLL-2 cells caused a significant increase in the expression of PD-1 and TIM-3 (Figure 3(e)), whereas PRF1 and GzmB protein levels decreased (Figure 3(f)). In contrast to TM, 4-PBA induced a reduction in PD-1 and TIM-3 expression levels, whereas PRF1 and GzmB protein levels increased markedly (Figures 3(g) and 3(h)). These data indicated that depletion of cells is associated with ER stress.

### 3.4. ER Stress Induces Cellular Depletion through the PERK-CHOP Pathway

**Figure 4:**
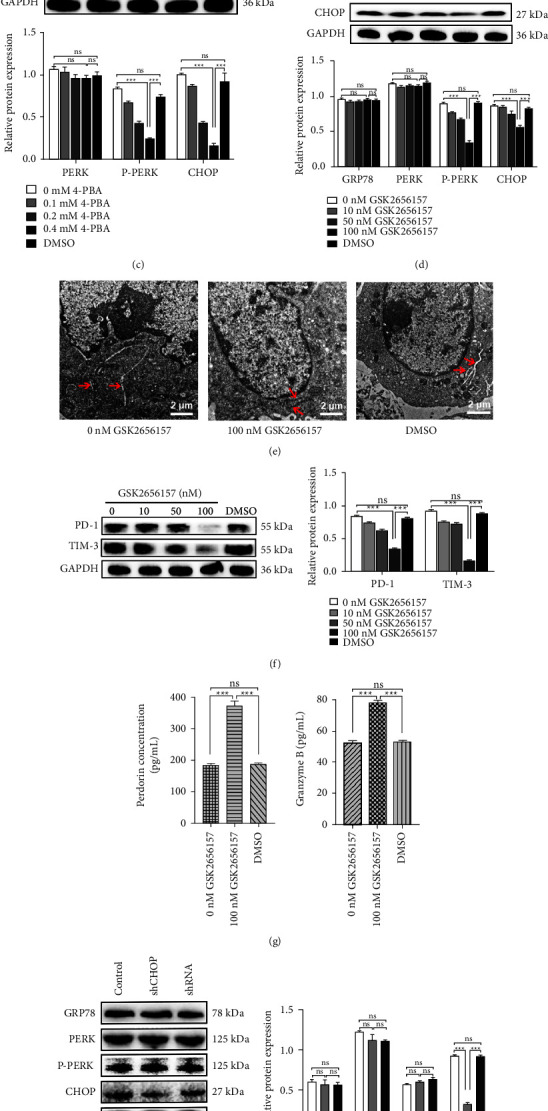
CTLs depletion was induced through the ER stress PERK-CHOP pathway. After coculturing hepatoma cells with CTLL-2 cells, (a) western blotting was used to examine the expression of PERK, P-PERK, and CHOP. (b) TM-induced protein expression of PERK, P-PERK, and CHOP. (c) 4-PBA treatment induced a reduction in PERK, P-PERK, and CHOP protein levels. (d) Cells were cocultured for one day and CTLL-2 cells were treated with either GSK2656157 (0, 10, 50 and 100 nM) for one day. Western blotting analysis of ER stress-associated protein expression. (e) Electron microscopic analysis (scale bar = 2 *μ*m). (f) Protein expression of PD-1 and TIM-3 in CTLL-2 cells. (g) Expression of PRF1 and GzmB in CTLL-2 cells. (h) Expression of ER stress-associated proteins in CTLL-2 cells after one day of silencing CHOP plasmid (0 and 0.25 *μ*g/*μ*L) treatment. (i) ER morphology was detected by electron microscopy (scale bar = 2 *μ*m). (j) Western blotting and (k) ELISA analysis. Data are presented as the means ± SD. ^*∗∗∗*^*P* < 0.001, ns indicates no statistical significance.

There are many pathways for ER stress development; among these pathways is the PERK-CHOP pathway, which plays an important role in ER stress [5,17]. Thus, we examined whether ER stress occurred in CTLL-2 cells via the PERK-CHOP pathway. Initially, western blotting analyses revealed that P-PERK and CHOP expression increased in the coculture of CTLL-2 cells and Hepa1-6 cells (Figure 4(a)). Following TM stimulation, the expression of ER stress-related proteins P-PERK and CHOP was increased significantly (Figure 4(b)). In contrast, 4-PBA treatment caused a reduction in protein levels of P-PERK and CHOP (Figure 4(c)). The results demonstrate that ER stress occurs through the PERK-CHOP pathway. Then, to examine whether cell depletion is induced by the PERK-CHOP pathway active in the ER, cells were treated with the PERK inhibitor GSK2656157 or silencing CHOP plasmid. We examined the levels of P-PERK and CHOP in CTLL-2 cells and found that the expression of these two proteins was downregulated significantly (Figure 4(d)), the ER returned to a normal morphology (Figure 4(e)), the expression of PD-1 and TIM-3 decreased (Figure 4(f)), and the protein levels of PRF1 and GzmB increased (Figure 4(g)). Similar results were obtained when cells were treated with the silencing CHOP plasmid (Figures 4(h)–4(k)). These results further confirmed that CTLs undergoing ER stress lead to a depletion in cell numbers through the PERK-CHOP pathway.

### 3.5. Inhibition of ER Stress by TP Reverses CTLs Depletion

**Figure 5:**
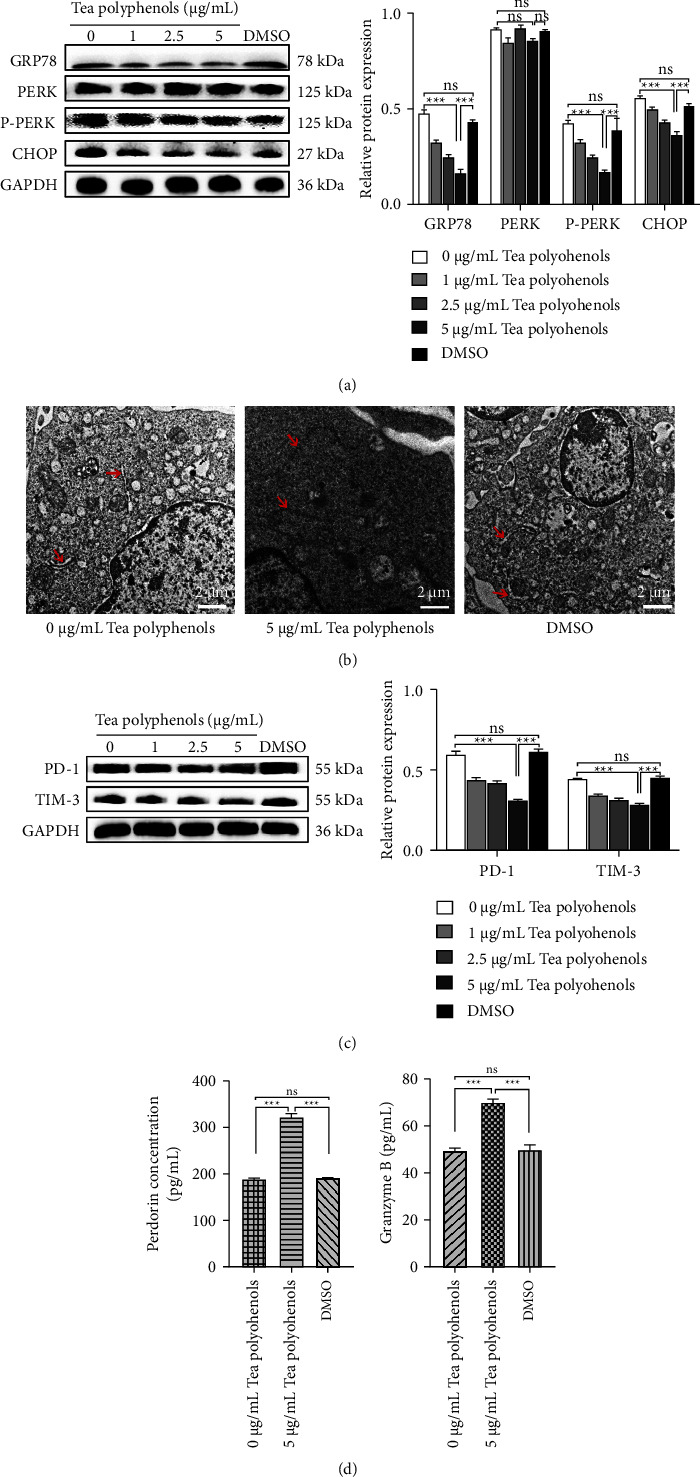
The reversal of CTLs depletion after treatment with TP. After the cells were cocultured for one day, different concentrations of tea polyphenols (0, 1, 2.5, and 5 *μ*g/mL) were added, and the cells were cultured for an additional day. (a) Western blotting analysis of GRP78, PERK, P-PERK, and CHOP expression in CTLL-2 cells. (b) Cells were treated with TP (0 and 5 *μ*g/mL), and ER morphology was examined by electron microscopy (scale bar = 2 *μ*m). (c) Western blotting and (d) ELISA analysis. Data are presented as the mean ± SD. ^*∗∗∗*^*P* < 0.001, ns indicates no statistical significance.

Because TP inhibit the growth of tumor cells [18,19], we hypothesized that tumor growth may be inhibited with tea polyphenol-regulated tumor-inducing CTLL-2 cells. To test this hypothesis, we revealed low expression of ER stress-related proteins GRP78, P-PERK, and CHOP in CTLL-2 cells after adding TP (Figure 5(a)). Moreover, these tea polyphenol-treated CTLL-2 cells displayed normal ER morphology (Figure 5(b)), a reduction in PD-1 and TIM-3 expression levels (Figure 5(c)), and an increase in PRF1 and GzmB protein levels (Figure 5(d)). The experiment revealed that TP inhibited ER stress and reversed cell depletion, providing a potential new approach to treating liver cancer.

## 4. Discussion

Multiple external factors and cell-intrinsic events can disrupt the protein folding activity of the ER. For example, tumor antigen stimulation causes an accumulation of misfolded or unfolded proteins, which provokes ER stress. [20, 21] In this study, we showed marked swelling of the ER and upregulation of ER stress-related protein GRP78 in CTLL-2 cells after coculturing with tumor cells. These observations are supported by a recent study revealing that ER stress is linked to immune cell function, [22] and Liu et al. [23] showing that ER-stressed HCC cells release exosomes to upregulate PD-L1 expression in macrophages, which subsequently inhibits T-cell function through an exosome miR-23a-PTEN-AKT pathway. We investigated the relationship between ER stress triggered by hepatoma cells and CTLL-2 cells and cellular function. We revealed that administration of TM, an ER stress activator, induced ER stress in CTLL-2 cells, with GRP78 protein levels upregulated significantly and noticeable swelling of the ER. Moreover, protein expression of PD-1 and TIM-3 increased, whereas PRF1 and GzmB protein expression decreased, suggesting that cellular functions were downregulated. TM-induced effects of the ER on cell function are consistent with the action of 4-PBA. Thus, we clarified that CTLL-2 cell depletion was caused by ER stress.

ER stress can stimulate cellular unfolded protein response (UPR), which initiates the expression of GRP78. Then, three pathways are activated, including IRE1, PERK, and ATF6, [20, 24] and the PERK pathway is a major branch of the UPR. Activated PERK proteins enhance the transcription of CHOP proteins. [25] Our data herein showed that ER stress occurred in the coculture group, and the levels of ER stress-related proteins P-PERK and CHOP increased significantly. After treatment with the PERK inhibitor and silencing CHOP plasmid, the expression of ER stress-related proteins decreased, and the ER morphology returned to normal. Concurrently, the detection of cell function indicators revealed that the expression of PD-1 and TIM-3 decreased, whereas the levels of PRF1 and GzmB increased. These results suggest that ER stress is activated through the PERK-CHOP pathway and leads to cellular depletion.

HCC has multiple forms of treatment, such as molecular targeted agents which, although much progress has been made, have a very poor prognosis due to drug resistance and frequent recurrence and metastasis [26]. Recently, the treatment of cancer by reversing T cell depletion is a hot topic in clinical research, and its function can be improved by blocking the PD-1 and TIM-3 pathways, [27] however, there are several unfavorable side effects to this approach. Interestingly, reports suggest that many herbs such as TP exert anticancer effects by inhibiting ER stress pathways in various cancers [28, 29]. But the role of TP in T cells has not been reported. Our experiments showed that TP decreased the expression of GRP78, P-PERK, and CHOP in CTLL-2 cells, and the ER morphology returned to normal, indicating that TP inhibit ER stress. In addition, detection of PD-1 and TIM-3 and PRF1 and GzmB expression suggest that TP treatment enhanced the cellular function. These findings indicate that TP reversed CTLL-2 cells depletion by inhibiting ER stress.

In summary, we found that CTLL-2 cells undergo ER stress that causes cell depletion through the PERK-CHOP pathway, and ER stress and restoration of cell functions can be partly reversed by adding TP, which provides a potential new therapeutic approach to treat liver cancer.

## 5. Conclusions

Hepatocellular carcinoma cells caused ER stress after coculturing with CTLL-2 cells and induced cellular disorders through the PERK-CHOP pathway. TP inhibited ER stress, and therefore, reversed cellular depletion.

## Data Availability

The data used to support the findings of this study are available from the corresponding author upon request.
